# A feasibility study of mindfulness-based interventions for children

**DOI:** 10.1186/s40814-024-01488-2

**Published:** 2024-04-08

**Authors:** Wai Man Sin, Mimi Mun Yee Tse, Joanne Wai Yee Chung, Sandy Pin Pin Choi

**Affiliations:** 1 School of Nursing and Health Studies, Hong Kong Metropolitan University, Kowloon, Hong Kong; 2 Guangzhou Medical University, Guangzhou, China; 3 Kiang Wu Nursing College of Macau, Macao, China

**Keywords:** Mindfulness interventions, Children’s well-being, Psychological wellness

## Abstract

**Background:**

Children’s overall psychological well-being is a concern for parents and adults worldwide. Mindfulness appears to be a promising intervention for enhancing children’s psychological well-being, and its effectiveness has been well-documented. However, there is a paucity of data on the feasibility and acceptability of implementing mindfulness-based interventions (MBIs) for children; this is a crucial factor in determining whether MBIs can be utilized to benefit children. The aim of this study was to determine the feasibility and acceptability of implementing MBIs among Hong Kong children.

**Methods:**

Seventy-eight children (mean age = 9.06, SD = .375) were recruited from a primary school in Hong Kong and received MBIs in a single session that lasted about 2 h. The intervention’s feasibility was determined in terms of retention rates, while acceptability was based on qualitative feedback from the children.

**Results:**

The results show that there were high retention rates (96%). Qualitative analyses of children’s feedback revealed that they experienced enhanced well-being, and enjoyed and benefited from the interventions.

**Conclusions:**

This study shows the high feasibility of MBIs in children, supporting the conduct of an efficacy trial to examine the effects of MBIs among children. Support from school teachers and measures to raise and maintain children’s interest in mindfulness could facilitate the conduct of a study.

## Key messages regarding feasibility

- Little is known about whether mindfulness-based interventions are feasible and acceptable for children.
- This study offers evidence to support the feasibility and acceptability of mindfulness-based interventions for children, as participants experienced enhanced well-being, and enjoyed and benefited from the interventions.
- Mindfulness-based interventions could be incorporated into the existing mental health services for children to serve as preventive measures.
- Support from school teachers and raising and maintaining children’s interest in mindfulness are crucial to the successful implementation of MBIs in schools.

## Background

A recent study revealed that almost one in ten Hong Kong children have poor psychological well-being [1]. Psychological well-being can be conceptualized as an overarching construct that encompasses six dimensions: self-acceptance, positive relations with others, autonomy, environmental mastery, purpose in life, and personal growth [2, 3]. Stress arising from pursuing academic excellence to live up to parental expectations has been identified as a key factor accounting for poor psychological well-being among children in Hong Kong [4, 5]. Consistent findings indicate that children experiencing poor psychological well-being are more likely to develop physical and developmental health problems [6]. When left untreated, it can have long-term negative mental and physical health implications, including an increased risk of developing mental illness and functional impairment in adulthood [7].

To date, the Hong Kong government has initiated mental health services at different levels, including annual assessments of children’s psychological well-being at Student Health Service Centers, providing primary school teachers with mental health training programs, and subsidizing primary schools to obtain additional resources, in pursuit of early detection and treatment of children who are at risk or who are already suffering from psychological problems [8]. However, these services have been criticized for focusing on detection and treatment, amid the increasing global consensus of rather shifting the focus of mental health services for children from detection and treatment to prevention [8]. To this end, it is crucial to identify an intervention that is effective in promoting children’s psychological well-being and incorporate it into the existing mental health services for children to serve as a preventive measure.

Mindfulness shows promise in this regard. The concept of mindfulness has been conceptualized as being aware of and openly attending to experiences, emotions, and thoughts in the present moment [9, 10]. Mindfulness can be cultivated through structured programs, such as mindfulness-based stress reduction programs, or brief interventions, such as slow controlled breathing and guided imagery. Regardless of the type of intervention, those that are primarily designed and used to cultivate mindfulness are regarded as mindfulness-based interventions (MBIs) [9]. Several empirical studies have examined MBIs for children, of which the majority focused on its effectiveness [11–14] while leaving its feasibility and acceptability relatively unexplored. It has been suggested that data on these aspects are essential to determine the fit of the interventions among the target group; therefore, more research is required prior to their implementation in real-world settings [15].

Although studies exploring the feasibility and acceptability of MBIs among children [16–18] have promising findings, their validity is limited by questionable sample sizes. There is no information on how the sample size was determined, but it was questioned if the sample size was large enough to reject the hypothesis [17, 18]. Further, these studies did not clearly state whether the criteria used to justify MBIs were feasible; however, the latest guideline for pilot and feasibility trials recommended that feasibility studies should specifically indicate the prespecified criteria employed to determine whether the studies/interventions are feasible and can proceed with a future larger-scale trial [19].

In light of these restrictions, the aim of this study was to examine the feasibility and acceptability of MBIs for children using a sample size determined based on a selected feasibility outcome (retention rate). The progression criteria for feasibility in this study was the retention rate. With reference to prior studies examining the feasibility of interventions among children [17, 20], if “not feasible” was set as a retention rate ≤ 50%, further studies should not be conducted; if “feasible” was defined as a retention rate ≥ 70%, larger-scale trials could be conducted [21].

## Methods

### Participants

The participants were children who were recruited from a primary school through convenience sampling. Those who were in Primary Four (approximately age 9–10) and were able to communicate in Cantonese were included in the study. There were no exclusion criteria.

The sample size calculation was based on the framework and approach to sample size derivation proposed by Lewis et al. [21] and focused on feasibility outcome (retention rate). Given a retention rate ≤ 50% (not feasible) and ≥ 70% (feasible), the number of participants required to reject the hypothesis with a power of 0.80 and an alpha of 0.05 (one-tailed) based on normal approximation was 73.

### Procedures

The study procedure was approved by the Human Research Ethics Committee of the Education University of Hong Kong (Reference number: 2018–2019-0193). Participant recruitment commenced in early October and was completed in mid-October, 2019. Participants were first nominated by the school liaison of the primary school and then screened by the first author. If the eligibility criteria were fulfilled, invitations to participate, together with the consent forms and information sheets, were sent to the parents/guardians of the participants. The option of withdrawing their children from the study at any time was emphasized. Informed consent from both participants and their parents/guardians was sought.

### Intervention

Four MBIs were delivered and included: (a) bell of mindfulness (used bell and inviter which is a short wooden rod to produce sound and encouraged participants to follow the sound of the bell); (b) mindful breathing (instructed participants to pay attention to their breath); (c) mindful walking (participants were led to the sports ground of the school; once there, they were given a bowl of water and instructed to be aware of how the water moves when they walked); and (d) mindful eating (participants were given marshmallows and encouraged to focus on the sensation, feelings, and thoughts they experience when they ate the marshmallow). All these interventions were suggested to help bring awareness back to the present moment and become non-judgmentally aware of the feelings, thoughts, and emotions at the moment [9, 22].

These interventions were tailored according to the children’s developmental and cognitive levels, and strategies were adapted to get participants engaged in the session and help them achieve mindfulness. The strategies included using simple phrases to guide participants to practice mindfulness, making the practices enjoyable (such as eating marshmallows, and food that most children like, during mindful eating), and shortening the duration of each practice [23].

Participants were divided into three groups according to their classes to keep the groups manageable and to ensure that the interventions were delivered to each participant. The interventions took place during the school day. One 2-h session was provided to each group. All MBIs were delivered in activity rooms/sports grounds of the school, from October 2019 to December 2019, by a qualified trainer in mindful self-compassion and mindfulness-based stress reduction.

We adopted certain measures to ensure the participants’ safety during interventions. First, in addition to the trainer, there were several assistants who helped to maintain class order, address participants’ needs, and ensure participants’ safety. Second, the school liaison was present in all sessions and would be informed to take appropriate actions, such as referral to the school counselor, in case the participants were at risk of developing/developing adverse reactions, such as feelings of depression.

### Outcome measures

#### Feasibility

The feasibility outcome was the retention rate which was operationalized as the total number of participants who completed the questionnaires post-intervention divided by those at baseline [24]. As aforementioned, the retention rate of ≥ 70% was regarded as feasible.

#### Acceptability

The acceptability of MBIs was determined based on qualitative feedback from participants [25–27]. Focus group interviews in a semi-structured format were conducted after the interventions. Participants were randomly selected using computer-generated numbers and divided into groups. Each group consisted of six to eight participants and was interviewed for around 30 min. The participants were asked a series of questions and led into a discussion. The questions were developed in accordance with the research question of this study (examining acceptability) and based on similar studies examining the topic [28, 29]. In addition to considering children’s shorter attention spans, a total of six questions were developed, as shown in Table 1. Probes were also set to elicit more details from them. All interviews were led by the first author who has received research training at the postgraduate level and conducted at the primary school during school hours.

**Table 1:** Focus group questions

Focus group questions
1. Please introduce yourself.
2. How would you describe your current well-being? Probes: from physical, psychological, cognitive, spiritual, and social perspectives
3. What strategies would you adopt previously/currently to restore your well-being? Probes: After learning MBIs, did you adopt these interventions to improve well-being when you encounter difficulties in your life? If yes, how often? Which MBIs would you adopt?
4.After the session and learning MBIs, did you regularly do these mindfulness practices? Probes: If yes, when? Which MBIs did you practice? Which MBIs help you the most?
5. Could you tell me of your experiences/feelings when practicing MBIs?
6. What else did you want to tell me about the MBIs?

### Data analyses

Prior to data analyses, the data were checked for completion and accuracy and screened for normality, outliers, and missing values. As a rule of thumb, less than 5% of missing values in the whole dataset was acceptable and would be included in the analyses [30]. Otherwise, the missing values would be addressed using multiple imputations.

The feasibility of adopting MBIs for children was determined in terms of retention rate and expressed as a percentage. For qualitative data obtained during focus group interviews to explore the acceptability of MBIs, the contents of the focus group interviews were first transcribed verbatim. A thematic analysis with an inductive approach was then utilized to identify, analyze, and report on the themes that emerged from these data. First, the researcher read the interview transcripts repeatedly, until they were familiar with the data. Parts of the data that were potentially salient to the research question (examined acceptability) were identified and labeled with codes. These codes relating to the same issue were categorized to form themes. The themes identified were reviewed to determine if they were supported with adequate data and distinct enough to differentiate from each other. Revisions continued until all identified data were appropriately categorized to support the themes, the themes identified were adequately supported with data, coherent but distinct from each other, and further refinement did not yield something new, meaning that saturation was reached. The themes were then named and data that were representative of the themes were extracted and presented, to illustrate the key meaning underlying the themes [31–33].

Descriptive statistics were used to summarize participants’ demographic characteristics. The findings were expressed as means and standard deviations (SD) for continuous variables and as frequencies and percentages for categorical ones.

## Results

### Demographic characteristics

Table 2 shows the demographic characteristics of participants at baseline. A total of 78 participants were enrolled in the study. The mean age of the participants was 9.06 years (SD = 0.375). More than half (56.4%) were male and did not have experience practicing mindfulness (55.1%).

**Table 2:** Demographic characteristics of participants at baseline (*N* = 78)

Characteristics	
Age–M (SD)	9.06 (.375)
	Number (%)
Gender	
Female	34 (43.6)
Male	44 (56.4)
Ethnicity	
Chinese	76 (97.4)
Religion	
Atheist	61 (78.2)
Buddhism	1 (1.3)
Catholic	1 (1.3)
Christian	8 (10.3)
Past medical history	
None	73 (93.6)
Asthma	3 (3.8)
Experience of practicing mindfulness	
No	43 (55.1)
Yes	29 (37.2)
Years of practicing mindfulness	
Less than 1 year	26 (33.3)
1–5 years	2 (2.6)
6–10 years	1 (1.3)
More than 10 years	1 (1.3)
Not applicable	40 (51.3)
Hours of practicing mindfulness per week	
Less than 1 h	24 (30.8)
1–3 h	4 (5.1)
4–6 h	2 (2.6)
Not applicable	40 (51.3)

### Feasibility

Of the 78 participants who enrolled, 75 completed the questionnaires post-intervention—a retention rate of 96%.

### Acceptability

A total of 42 participants were involved in the focus group interviews. The thematic analysis, performed using the children’s responses during the interviews, revealed two main themes related to the research question (examine acceptability). Table 3 presents these identified themes and subthemes.

**Table 3:** Themes and subthemes analyzed from children’s responses

Themes		Subthemes
Theme 1:	Changes experienced after practicing mindfulness	1. Psychological aspects 2. Cognitive aspects
Theme 2:	Utilization of mindfulness	1. Prompts to use mindfulness 2. Types of mindfulness practice

#### Theme 1: changes experienced after practicing mindfulness

Recurring comments were received from the children indicating that they experienced changes after practicing mindfulness. These changes were categorized into two subthemes: psychological and cognitive aspects. Psychological aspects were the stronger of the two subthemes. More than half of the children reflected on experiencing more positive emotions after practicing mindfulness. Moreover, most noticed that mindfulness could boost their feelings of happiness, whereas a few expressed having some relief from stress after using mindfulness.

Child 1: “I felt good and very happy after doing mindful breathing.”

Child 2: “I experienced much relief and less stress after practicing mindfulness.”

Changes were also noticeable in other aspects, including improved sleep quality and a better ability to control their behavior.

Child 1: “I can now fall asleep more easily, as mindfulness helped in clearing the mind and stopping rumination.”

Child 2: “After doing mindful breathing, I had a clearer idea of what I should do and had better ability to control my temper, instead of venting the emotion in unhealthy ways.”

#### Theme 2: utilization of mindfulness

The second theme drawn from the responses was the utilization of mindfulness. Within this theme, two subthemes emerged: (1) prompts to use mindfulness and (2) types of mindfulness practice. The frequency with which the children reported performing mindfulness varied, from practicing it every day to never doing it. The situation prompting them to engage in mindfulness was most often when they were experiencing negative emotions. Five children recalled using mindfulness when they got angry. Another four children remarked that they would perform mindfulness on occasions when feeling unhappy.

Interviewer: “When did you do mindfulness?”

Child 1: “When I felt angry.”

Child 2: “When I felt sad.”

Child 3: “When I got poor examination results.”

Another moment that the children would think of practicing mindfulness was when they encountered stressful situations. These situations were mostly related to their studies.

Interviewer: “When did you do mindfulness?”

Child 1: “When I felt nervous during the dictations/examination.”

Child 2: “When I couldn’t understand what the teachers taught during lessons.”

When asked which mindfulness practice they commonly used and found helpful in these situations, nearly all the children chose mindful breathing.

Interviewer: “Which mindfulness practice helped you the most?”

All the children in the focus group reported at the same time: “Mindful breathing.”

The most impressive mindfulness practices were those integrated with “fun” or “game” elements. Approximately one-sixth of the children reported that they remembered mindful eating because they were provided with marshmallows during mindful eating. Some of them (four children) excitingly shared their experiences with mindful breathing because dolls were placed on their tummies during mindful breathing.

Child 1: “I learned how to eat the marshmallow.”

Child 2: “I laid down, and a doll was placed on my tummy.”

## Discussion

This study explored the feasibility and acceptability of implementing MBIs for children in preparation for a larger-scale trial. Findings from this study imply that implementing MBIs is feasible and acceptable as reflected by the high retention rate and the fact that children continued practicing mindfulness on their own even after the sessions.

A retention rate of 96% was found in the current study, which is considerably higher than the lower bound of “feasible” that we set. Based on this, we suggest that MBIs are feasible for children. This was possibly promoted by ensuring convenience for the children. Both interventions and data collection were conducted at children’s schools during school hours; therefore, children were more likely to join and remain part of the study. More importantly, obtaining support from the school teachers is likely the key to the high retention rate. During the present study, apart from providing coordination and assistance in both interventions and data collection, they also helped explain the study and clarify information to the students and their parents. Teachers are usually the first person students/their parents would talk to in case of any concern; therefore, students/their parents were more likely to join and continue in the study. We employed two strategies to gain support from school teachers. First, we clearly described the aims, procedures, and potential benefits of practicing mindfulness to school teachers. It was suggested that school teachers would be more willing to provide coordination and support when they believed the interventions were beneficial to the students [34]. Second, given that schools are primarily for teaching, it is understandable that any activity disturbing the school schedules and teaching is unlikely to be supported by school teachers. In this regard, we discussed the arrangement of the study with the school liaison ensuring that both interventions and data collection fitted the school schedules and did not interrupt them. In view of the importance of teachers’ support in a study, especially those conducted in schools, apart from the abovementioned strategies, other strategies to increase the likelihood of gaining support from school teachers should be employed in future studies. One potential strategy is to establish an effective channel to communicate with school teachers; in addition to traditional methods, new approaches, such as WhatsApp, are worth considering [34].

Our study also found that MBIs were acceptable to children, as indicated by the positive feedback and continuation of mindfulness practice after sessions. Children described that they experienced positive changes after practicing mindfulness; these included having more positive emotions, improved sleep quality, and increased ability to control their behavior. Besides, children were impressed with mindfulness practices, especially those integrated with “fun” or “game” elements. This finding is in contrast with that of a previous study, which reported that most participants did not show interest in learning and practicing mindfulness, as evidenced by a high dropout rate and low attendance rate [35]. The possible reason the researchers provided is the passive and quiet nature of mindfulness practices. Some participants even described the mindfulness practices as dull and boring [35]. This discrepancy in the findings highlights the need for different strategies in future studies and modifications to mindfulness interventions, such as the integration of “fun” elements when teaching children/adolescents mindfulness to raise their interest. Raising their interest in mindfulness is identified as the first and most crucial step to keep them practicing mindfulness [35]. Once children begin to practice mindfulness regularly, integrate it into their daily living, and make it a habit, they will benefit the most [36].

## Limitations and future directions

The present study had three main limitations. The first limitation was that only one feasibility outcome (retention rate) was used and one area of focus (acceptability) was explored. In the future, it is important to employ more feasibility outcomes, such as a recruitment rate, and explore other areas of focus, such as adaptation [25] to examine the feasibility of MBIs among children more comprehensively. Another limitation was that the feasibility data and perspectives were only sought from children. Considering that school executives and teachers play crucial roles in schools, future research exploring their views on the feasibility of MBIs among children is warranted.

## Conclusion

The findings of this study add to the growing body of literature supporting the feasibility and acceptability of MBIs for children. Future trials would benefit from employing more feasibility outcomes and exploring the feasibility from different perspectives. Gaining support from schoolteachers and raising and sustaining children’s interest in mindfulness can optimize the conduct of a study.

## Data Availability

The datasets used and/ or analyzed in the present study are available from the corresponding author on reasonable request.

## Abbreviations

MBI: Mindfulness-based interventions
SD: Standard deviation

## References

[1] Rees G, Savahl S, Lee BJ, Casas F. Children’s views on their lives and well-being in 35 countries: a report on the children’s worlds project, 2016–19. 2020. https://isciweb.org/wp-content/uploads/2020/08/Childrens-Worlds-Comparative-Report-2020.pdf. Accessed 26 Sep 2022.

[2] Burns R, Pachana NA (2016). Psychosocial well-being. Encyclopedia of geropsychology.

[3] Ryff CD, Singer B (1996). Psychological well-being: meaning, measurement, and implications for psychotherapy research. Psychother Psychosom.

[4] Hung J (2018). Parenting styles, academic demands and children’s psychosocial well-being: why today’s Hong Kong Chinese students are so stressed. J Glob Health.

[5] Haden-Pawlowski V, Pui C, Ng E, Li L, Law C. Mental health matters – save the children in Hong Kong – 2020. 2020. https://www.researchgate.net/publication/346520015_Mental_Health_Matters_-_Save_the_Children_Hong_Kong_-_2020. Accessed 26 Sep 2022.

[6] Local Government Association. Children and young people’s emotional wellbeing and mental health – facts and figures. [date unknown]. https://www.local.gov.uk/about/campaigns/bright-futures/bright-futures-camhs/child-and-adolescent-mental-health-and. Accessed 26 Sep 2022.

[7] Australian Government. The national children’s mental health and wellbeing strategy. [date unknown]. https://www.mentalhealthcommission.gov.au/getmedia/5b7112be-6402-4b23-919d-8fb9b6027506/National-Children%E2%80%99s-Mental-Health-and-Wellbeing-Strategy-%E2%80%93-Report. Accessed 26 Sep 2022.

[8] Lam C. Mental health services for young people. 2021. https://www.legco.gov.hk/research-publications/english/essentials-2021ise31-mental-health-services-for-young-people.htm. Accessed 26 Sep 2022.

[9] Creswell JD (2017). Mindfulness interventions. Annu Rev Psychol.

[10] Stahl  B, Goldstein  E (2010). A mindfulness-based stress reduction workbook.

[11] Crescentini C, Capurso V, Furlan S, Fabbro F (2016). Mindfulness-oriented meditation for primary school children: effects on attention and psychological well-being. Front Psychol..

[12] Malboeuf-Hurtubise C, Léger-Goodes T, Mageau GA, Joussemet M, Herba C, Chadi N (2021). Philosophy for children and mindfulness during COVID-19: results from a randomized cluster trial and impact on mental health in elementary school students. Prog Neuro-Psychopharmacol Biol Psychiatry.

[13] Joyce A, Etty-Leal J, Zazryn T, Hamilton A, Hassed C (2010). Exploring a mindfulness meditation program on the mental health of upper primary children: a pilot study. Adv Sch Ment Health Promot.

[14] van de Weijer-Bergsma E, Langenberg G, Brandsma R, Oort FJ, Bögels SM (2014). The effectiveness of a school-based mindfulness training as a program to prevent stress in elementary school children. Mindfulness.

[15] Wight D, Wimbush E, Jepson R, Doi L (2016). Six steps in quality intervention development (6SQuID). J Epidemiology Community Health..

[16] Blasi ZD, Rice A, Van Zyl LE, Rothmann S (2019). The effectiveness, feasibility and acceptability of a mindfulness-based intervention in two Irish primary schools. Evidence-based positive psychological interventions in multi-cultural contexts.

[17] Lee J, Semple RJ, Rosa D, Miller L (2008). Mindfulness-based cognitive therapy for children: results of a pilot study. J Cogn Psychother.

[18] Semple RJ, Reid EFG, Miller L (2005). Treating anxiety with mindfulness: an open trial of mindfulness training for anxious children. J Cogn Psychother.

[19] Eldridge SM, Chan CL, Campbell MJ, Bond CM, Hopewell S, Thabane L (2016). CONSORT 2010 statement: extension to randomised pilot and feasibility trials. BMJ.

[20] Wang LJ, Chen CK, Hu JF, Chiang LM, Shang ZY, Wang LY (2016). Outcomes and associated factors of children with developmental delay in an early intervention program. Taiwan J Psychiatry.

[21] Lewis M, Bromley K, Sutton CJ, McCray G, Myers HL, Lancaster GA (2021). Determining sample size for progression criteria for pragmatic pilot RCTs: the hypothesis test strikes back!. Pilot Feasibility Stud.

[22] Nhat Hạnh  T, Weare K (2017). Happy teachers change the world: a guide for cultivating mindfulness in education.

[23] Shonin E, Van Gordon W, Griffiths MD (2014). Practical tips for teaching mindfulness to children and adolescents in school-based settings. Educ Health.

[24] Rung AL, Oral E, Berghammer L, Peters ES (2020). Feasibility and acceptability of a mobile mindfulness meditation intervention among women: intervention study. JMIR mhealth uhealth.

[25] Bowen DJ, Kreuter M, Spring B, Cofta-Woerpel L, Linnan L, Weiner D (2009). How we design feasibility studies. Am J Prev Med.

[26] Giesbrecht EM, Miller WC (2017). A randomized control trial feasibility evaluation of an mHealth intervention for wheelchair skill training among middle-aged and older adults. PeerJ.

[27] Patel SJ, Subbiah S, Jones R, Muigai F, Rothschild CW, Omwodo L, et al. Providing support to pregnant women and new mothers through moderated whatsApp groups: a feasibility study. mHealth. 2018; 4(14). 10.21037/mhealth.2018.04.05 10.21037/mhealth.2018.04.05 PMC5994467 29963559

[28] Ager K, Albrecht N, Cohen M (2015). Mindfulness in school research project: exploring students’ perspectives of mindfulness – what are students’ perspectives of learning mindfulness practices at school?. Psychology.

[29] Bernay R, Graham E, Devcich DA, Rix G, Rubie-Davies CM (2016). Pause, breathe, smile: a mixed-methods study of student well-being following participation in an eight-week, locally developed mindfulness program in three New Zealand schools. Adv Sch Ment Health Promot.

[30] University of Prince Edward Island. Working with missing data. [date unknown]. https://pressbooks.library.upei.ca/montelpare/chapter/working-with-missing-data/. Accessed 26 Sep 2022.

[31] Bengtsson M (2016). How to plan and perform a qualitative study using content analysis. NursingPlus Open.

[32] Kiger ME, Varpio L (2020). Thematic analysis of qualitative data: AMEE guide No. 131. Med Teach.

[33] Korstjens I, Moser A (2018). Series: Practical guidance to qualitative research. Part 4: trustworthiness and publishing. Eur J Gen Pract..

[34] Schoeppe S, Oliver M, Badland HM, Burke M, Duncan MJ (2014). Recruitment and retention of children in behavioral health risk factor studies: REACH strategies. IJBM.

[35] Lam CC, Lau NS, Lo HH, Woo DMS (2015). Developing mindfulness programs for adolescents: Lessons learned from an attempt in Hong Kong. Soc Work Ment Health.

[36] Germer C, Neff K (2019). Teaching the mindful self-compassion program.

